# SIRT1-regulated ROS generation activates NMDAR2B phosphorylation to promote central sensitization and allodynia in a male chronic migraine rat model

**DOI:** 10.3389/fnmol.2024.1387481

**Published:** 2024-05-22

**Authors:** Xiaoyan Zhang, Wei Zhang, Yanyun Wang, Yun Zhang, Dunke Zhang, Guangcheng Qin, Jiying Zhou, Lixue Chen

**Affiliations:** ^1^Department of Neurology, The First Affiliated Hospital of Chongqing Medical University, Chongqing, China; ^2^Laboratory Research Center, The First Affiliated Hospital of Chongqing Medical University, Chongqing, China

**Keywords:** SIRT1, central sensitization, chronic migraine, reactive oxygen species (ROS), NMDAR2B phosphorylation

## Abstract

**Background:**

Central sensitization is one of the pivotal pathological mechanisms in chronic migraine (CM). Silent information regulator 1 (SIRT1) was shown to be involved in CM, but its specific mechanism is unclear. Reactive oxygen species (ROS) are increasingly regarded as important signaling molecules in several models of pain. However, studies about the role of ROS in the central sensitization of CM model are rare. We thus explored the specific process of SIRT1 involvement in the central sensitization of CM, focusing on the ROS pathway.

**Methods:**

Inflammatory soup was repeatedly administered to male Sprague–Dawley rats to establish a CM model. The SIRT1 expression level in trigeminal nucleus caudalis (TNC) tissues was assessed by qRT–PCR and Western blotting analysis. The levels of ROS were detected by a Tissue Reactive Oxygen Detection Kit, DHE staining, and the fluorescence signal intensity of 8-OHdG. A ROS scavenger (tempol), a SIRT1 activator (SRT1720), a SIRT1 inhibitor (EX527), and a mitochondrial fission inhibitor (Mdivi-1) were used to investigate the specific molecular mechanisms involved. NMDAR2B, CGRP, ERK, and mitochondrial fission-related protein were evaluated by Western blotting, and the CGRP level in frozen sections of the TNC was detected via immunofluorescence staining.

**Results:**

After repeated inflammatory soup infusion and successful establishment of the CM rat model, SIRT1 expression was found to be significantly reduced, accompanied by elevated ROS levels. Treatment with Tempol, SRT1720, or Mdivi-1 alleviated allodynia and reduced the increase in NMDAR2B phosphorylation and CGRP and ERK phosphorylation in the CM rat. In contrast, EX527 had the opposite effect in CM rat. SRT1720 and EX527 decreased and increased ROS levels, respectively, in CM rats, and tempol reversed the aggravating effect of EX527 in CM rats. Furthermore, the regulatory effect of SIRT1 on ROS may include the involvement of the mitochondrial fission protein DRP1.

**Conclusion:**

The results indicate the importance of SIRT1 in CM may be due to its role in regulating the production of ROS, which are involved in modulating central sensitization in CM. These findings could lead to new ideas for CM treatment with the use of SIRT1 agonists and antioxidants.

## Introduction

Migraine is a common neurological disorder with a high rate of disability and an enormous global burden (GBD 2016 Headache Collaborators, 2018; Steiner et al., 2018; Ferrari et al., 2022). Chronic migraine (CM) is defined as headache ≥15 days per month for more than 3 months and migraine attack ≥8 days per month, with the potential for comorbidities with other conditions (anxiety, insomnia, depression, irritable bowel syndrome, etc.), further aggravating the disease burden (Headache Classifcation Committee of the International Headache Society (IHS), 2018; Yin et al., 2021). Despite the damaging effects of CM, the mechanisms behind CM remain to be elucidated fully. One of the leading theories suggests that central sensitization of the trigeminal nucleus caudalis (TNC) plays a critical role in the pathogenesis of CM (Bigal et al., 2008; Woolf, 2011; Boyer et al., 2014; Goadsby et al., 2017; Su and Yu, 2018). Central sensitization is defined as an increase in synaptic efficacy and excitability of neurons in central nociceptive pathways triggered by nociceptor inputs, results in a lower nociceptive threshold, an increased pain response, and spreading of pain sensitivity to non-injured areas (Ji et al., 2003; Woolf, 2011).

Several clinical studies have reported that migraine and CM patients have lower total serum antioxidant capacity and elevated indicators of oxidative stress, and migraine may be related to an increase in susceptibility to oxidative stress (Ciancarelli et al., 2007; Lucchesi et al., 2015; Geyik et al., 2016). Reactive oxygen species (ROS), as major substances engaged in oxidative stress, play pivotal roles in maintaining redox balance. ROS have been demonstrated to be involved in a variety of neurological disorders, including Alzheimer’s disease, Parkinson’s disease, dementia, cerebral ischemia, and stroke (Jung et al., 2016; Cheng et al., 2022; Du et al., 2022; Esteras et al., 2022; Zeng et al., 2022). Overproduction of ROS has also been implicated in several animal models of pain (Kim et al., 2004; Lee et al., 2012; Ilari et al., 2020; Shi et al., 2021; Yang et al., 2021), but we do not yet know whether ROS are involved in CM and its underlying mechanisms.

The N-methyl-D-aspartate receptor (NMDAR) is a glutamate receptor that forms one of the ion channel conductance pathways (Traynelis et al., 2010). NMDAR dysfunction contributes to a variety of neurological disorders and psychiatric disorders, such as cerebral ischemic injury, schizophrenia, depression, and cognitive impairment (Ferreira et al., 2017; Luo et al., 2018; Adell, 2020; Companys-Alemany et al., 2020). A great deal of evidence has demonstrated that NMDARs also play a significant role in nociceptive sensitization (Liu et al., 2008), particularly for NMDAR2B (NR2B), whose alterations include overexpression and altered phosphorylation status of GluN2B (Paoletti et al., 2013). Our previous study showed that a CM rat model involves changes in synaptic plasticity due to NR2B phosphorylation, which leads to hypersensitivity in the central nociceptive system (Wang et al., 2018). Additional studies have shown that ROS could be responsible for chronic pain by regulating the phosphorylation of glutamate ion channels (Lee et al., 2012; Ye et al., 2016). However, whether ROS are engaged in regulating the phosphorylation of NR2B and thereby contributing to central sensitization in CM models is unclear.

Silent information regulator 1, a member of the sirtuin family, is involved in multiple physiological and pathological processes, such as aging, metabolism, inflammation, and neuropathic pain (Chang and Guarente, 2014; Zhang et al., 2019; Xu et al., 2020; Wen et al., 2021). SIRT1 has also been reported to be involved in the regulation of mitochondrial fusion and fission by modulating mitochondrial dynamics-related proteins (Li et al., 2019). Mitochondria are important sources of ROS produced in the nervous system, and excessive mitochondrial fission further increases ROS production (Yu et al., 2006; Kishida and Klann, 2007; Kanda et al., 2016). Previous findings from our group have shown that SIRT1 expression is reduced in CM animal models (Liang et al., 2021; Wen et al., 2021). We therefore hypothesized that SIRT1 could be involved in CM by regulating mitochondrial fission proteins to induce ROS production, thereby mediating NR2B phosphorylation and central sensitization.

Based on these hypotheses, this study first evaluated SIRT1 and ROS levels in CM rat model and identified the influence of ROS scavenger on central sensitization and anti-allodynia in CM. We further validated the role of SIRT1 in CM and investigated the potential mechanisms, focusing mainly on ROS. Our results showed that SIRT1 was downregulated in CM rats, which led to allodynia by modulating the phosphorylation of NR2B via ROS.

## Materials and methods

### Animals

To minimize the multifactorial effects of sex and weight, adult male Sprague–Dawley (SD) rats weighing 250–300 g (specific pathogen-free) were used in our study. The SD rats were acquired from the Experimental Animal Center of Chongqing Medical University (China). The rats were kept in the laboratory at a constant temperature (24 ± 1°C) with food and water available *ad libitum* under a 12/12-h light/dark cycle. We made all efforts to minimize the rats’ number and to alleviate the suffering of the rats used in the experiments. Rats were randomly placed in the experimental groups after a 1-week habituation period. All care and experimental procedures on animals were in accordance with the National Institutes of Health Guide for the Care and Use of Laboratory Animals.

### Surgical procedures for the CM model

The surgical procedures for establishing the CM rat model were performed as described previously (Melo-Carrillo and Lopez-Avila, 2013). Before surgery, the rats were fasted for 8 h. The rats were first placed in a stereotaxic instrument (ST-51603; Stoelting Co., United States) following general anesthesia with sodium pentobarbital, (50 mg/kg, intraperitoneal injection). The area of surgery was sterilized with iodophor, and a 1–1.5 cm long vertical incision was made along the midline of the head. A hole was drilled in the head (the coordinates were − 1.0 mm posterior to the bregma and 1.5 mm lateral to the midline), and a capped sterile cannula (23-gage) was used to indwell and fixed outside the dura mater with dental cement (no dura mater damage). The rats were allowed to rest and awaken on an electric blanket (37°C). The rats were subsequently caged alone for 7 days for recovery, while the surgical area was sterilized daily with iodophor. Finally, 5 μL of inflammatory soup (IS) was infused into the dura through a cannula once a day for 7 days to establish the CM rat model, while sterile PBS was infused into the sham rats. The IS consists of 0.1 mM prostaglandin E2, 1 mM serotonin, 1 mM bradykinin, and 1 mM histamine (Sigma-Aldrich, St. Louis, MO, United States) in PBS.

### Animal groups and drug administration

For the purpose of the experiment, rats were randomized to the following groups: sham group, CM group, sham + DMSO group, CM + DMSO group, sham + Tempol group, CM + Tempol group, sham + SRT1720 group, CM + SRT1720 group, sham + EX527 group, CM + EX527 group, CM + EX527 + DMSO group, CM + EX527 + Tempol group, sham + Mdivi-1 group, and CM + Mdivi-1 group. All of the above drugs were injected 24 h after the 7-day IS/PBS infusion. The SRT1720 (10 μg), EX527 (1 and 10 μg), tempol (5 and 50 μg), and Mdivi-1 (3 μg), the doses of drugs used in these experiments were determined from previous studies (Yan et al., 2013; Dohare et al., 2014; Kanda et al., 2016; Liang et al., 2021). All the drugs were infused into the lateral ventricle (the coordinates were − 1.0 mm posterior to the bregma, 1.5 mm lateral to the midline, and 4 mm below the dura) in a volume of 5 μL over 10 min.

### Behavioral assays

Behavioral testing was carried out prior to the first IS/PBS infusion, 24 h after each IS/PBS infusion, and after each drug administration. The animal behavioral tests were conducted during daylight, and the experimenter conducting the behavioral testing was blinded to the groups. The rats were allowed to acclimatize to each testing platform for 30 min before testing to minimize transfer interference.

Cutaneous allodynia is an easy-to-identify central sensitization marker, and thermal sensitivity test and mechanical pain sensitivity test can be used to detect allodynia (Burstein et al., 2000; Romero-Reyes and Ye, 2013). The rats were placed in cages individually, and the areas of the periorbital and hind paw regions were applied with a von Frey monofilament (1–26 g) using the “up-down” method to measure the mechanical thresholds of the periorbital and hind paw, respectively (Chaplan et al., 1994; Romero-Reyes and Ye, 2013; Zeng et al., 2020). When the rat showed a withdrawal of the hind paw, head shaking, head scratching, licking of the hind paw, or head avoidance, it was regarded as a positive response.

The hind paw thermal threshold was conducted with a plantar test apparatus (Techman PL-200, China) as previously described (Zhang K. L. et al., 2022). Then, the rats were placed on the glass surface of a plate with a transparent chamber (27 cm × 27 cm × 27 cm), and infrared radiation at an intensity of 20% was aimed at the bottom of the hind paw. The radiation time (25 s maximum to prevent damage to the rat) was recorded automatically when the rat lifted its hind paw. The same test was performed three times, with more than 5 min between each test. The average of the tests was regarded as the hind paw thermal pain threshold.

### Quantitative real-time PCR

Trigeminal nucleus caudalis tissue was dissected quickly after each rat was sacrificed. RNA from TNC tissues was extracted using the RNAiso Plus reagent (TaKaRa, China). The RNA purity and concentration were measured with a NanoDrop spectrophotometer (Thermo, United States). The PrimeScript™ RT reagent Kit (TaKaRa, China) was used for the cDNA synthesis. To quantify the expression of SIRT1 mRNA, qRT–PCR was conducted on a CFX96 Touch thermocycler (Bio-Rad, United States) with SYBR Premix Ex Taq TM II (TaKaRa, China), and the mRNA expression of SIRT1 was normalized to GAPDH with the standard 2^−ΔΔCT^ method. The primer sequences involved are as follows:

SIRT1_forward (5′—3′): ACGCCTTATCCTCTAGTTCCTGTGG
SIRT1_reverse (5′—3′): CGGTCTGTCAGCATCATCTTCCAAG
GAPDH _forward (5′—3′): ATGACTCTACCCACGGCA AGCT
GAPDH _reverse (5′—3′): GGATGCAGGGATGATGTTCT

### Western blot analysis

Briefly, TNCs were harvested quickly after rats were sacrificed. Fresh TNC tissue was cleaved in radioimmunoprecipitation assay (RIPA) lysis buffer (Beyotime, China) containing proteinase inhibitors (Beyotime, China) and phosphatase inhibitors (MedChemExpress, United States). The concentration of proteins was subsequently measured with a BCA protein assay kit (Beyotime, China). A 10% SDS–PAGE gel was used to separate equal mass protein samples, after which the proteins were transferred to PVDF membranes. The PVDF membranes were incubated with primary antibodies at 4°C for one night. On the second day, the PVDF membranes were washed and incubated with the corresponding secondary antibodies for 1 h. After that, images were obtained under an imaging system (Fusion, Germany) with an ultra High Sensitivity ECL Kit (MedChemExpress, United States). In Table 1, all the primary and secondary antibodies used are listed.

**Table 1:** Antibodies used in western blotting and immunofluorescence analysis.

Antibody	Manufacturer	Host	Dilution
For western blot analysis			
SIRT1	Abcam, United Kingdom	Mouse	1:3,000
NR2B	Proteintech, United States	Rabbit	1:1,000
pNR2B-Y1472	CST, United States	Rabbit	1:1,000
CGRP	Abcam, United Kingdom	Rabbit	1:3,000
pERK	CST, United States	Rabbit	1:1,000
ERK	CST, United States	Rabbit	1:1,000
DRP1	Abcam, United Kingdom	Rabbit	1:1,000
β-Tubulin	ZEN-BIOSCIENCE, China	Mouse	1:6,000
Anti-rabbit IgG (HRP)	ZEN-BIOSCIENCE, China	Goat	1:5,000
Anti-mouse IgG (HRP)	ZEN-BIOSCIENCE, China	Goat	1:5,000
For immunofluorescence staining			
CGRP	Santa Cruz, United States	Mouse	1:100
8-OHdG	Santa Cruz, United States	Mouse	1:50
Alexa Fluor 488 goat anti-mouse IgG	Beyotime, China	Goat	1:400

### Immunofluorescence staining

After deep anesthesia with sodium pentobarbital (150 mg/kg, intraperitoneally), the rats were transcardially perfused with PBS, followed by 4% paraformaldehyde. TNC tissue was obtained, postfixed in 4% paraformaldehyde at 4°C for 12 h and cryoprotected with sucrose. A cryostat (Leica, Japan) was used to cut TNC tissue into 10 μm thick sections, and mounted sections on glass slides. The TNC sections were incubated with the primary antibodies and incubated at 4°C for 12 h. On the following day, the sections of TNC were incubated at 37°C for 1 h with the appropriate fluorescent secondary antibodies. A confocal microscope (Zeiss, Germany) was applied to acquire fluorescence images. For quantitative analysis, the immunofluorescence intensity was measured using ImageJ’s Analyze and Measure functions. The primary and secondary antibodies used for immunofluorescence are listed in Table 1.

### Tissue reactive oxygen species assay

The levels of tissue ROS were measured by the Tissue Reactive Oxygen Species Assay Kit (BestBio, China). The obtained TNC tissues were quickly washed with PBS, and 1 mL of homogenization buffer A was added to 50 mg of tissue, after which the mixture was homogenized. The homogenate was centrifuged (100 × *g*, 4°C) for 3 min, after which the supernatant was collected. 200 μL of the supernatant and 2 μL of DHE probe were added to a black 96-well plate and incubated at 37°C for 30 min in the dark. The fluorescence intensity was measured with an excitation wavelength of 510 nm and an emission wavelength of 610 nm. Finally, the supernatant homogenate was subjected to protein quantification, and the levels of ROS in the tissue was calculated as the fluorescence intensity/mg protein. Each sample was performed in triplicate.

### Dihydroethidium staining

The TNC slices were incubated with 10 mmol of DHE solution (Sigma–Aldrich, United States) for 30 min away from light, then the sections were washed three times with PBS. Images of the slices were obtained by observation under a confocal microscope (Zeiss, Germany).

### Statistical analysis

The data were statistically analyzed, and graphs were generated with GraphPad Prism version 8.0.1. Values are depicted as mean ± SEM. Statistical significance was assessed with the *t*-test, one-way ANOVA (followed by the Bonferroni *post hoc* test), or two-way ANOVA (followed by a Bonferroni *post hoc* test), depending on the proper design. *p* value <0.05 was considered as the threshold for significance. Statistical details of the experiments are depicted in each figure legend.

## Results

### SIRT1 expression decreased and ROS production increased in CM rats

**Figure 1:**
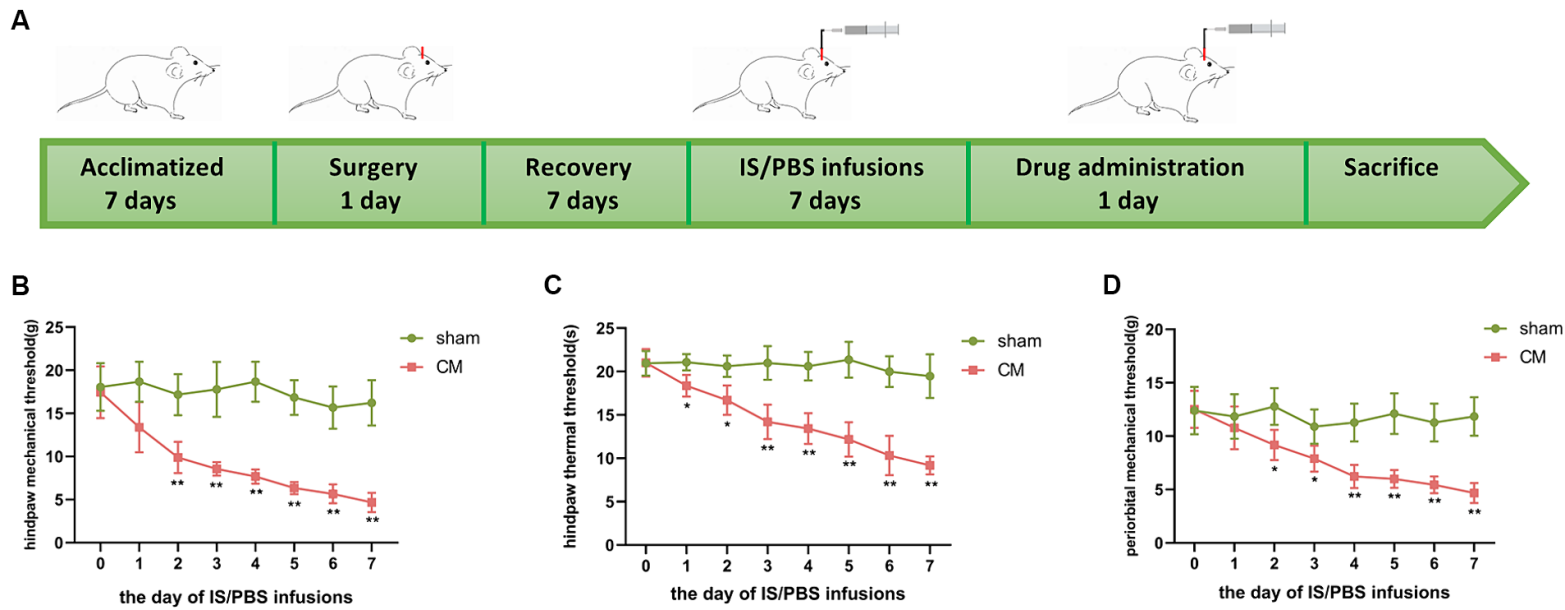
The design of animal experiments and changes in mechanical and thermal pain thresholds of rats. **(A)** The rats went through the stages of acclimatization, surgery, recovery, IS/PBS infusion, and drug administration sequentially. **(B)** Changes in hind paw mechanical thresholds in Sham and CM rats. **(C)** Changes in hind paw thermal thresholds in Sham and CM rats. **(D)** Changes in periorbital mechanical thresholds in Sham and CM rats. Two-way ANOVA with Bonferroni *post hoc* test. Data were expressed as mean ± SEM. ^*^p < 0.05, ^**^p < 0.01 vs. the Sham group; *n* = 6/group.

As shown in Figure 1A, the rats went through the stages of acclimatization, surgery, recovery, and PBS/IS infusion sequentially. The pain thresholds were tested on day 0 to day 7 of the daily infusion of PBS/IS into the dura mater to evaluate allodynia in sham rats and CM rats. As expected, the CM rats exhibited a significant reduction in the hind paw mechanical threshold, hind paw thermal threshold, and periorbital mechanical threshold, but no obvious changes were observed in the behavioral assays of the sham group (Figures 1B–D).

**Figure 2:**
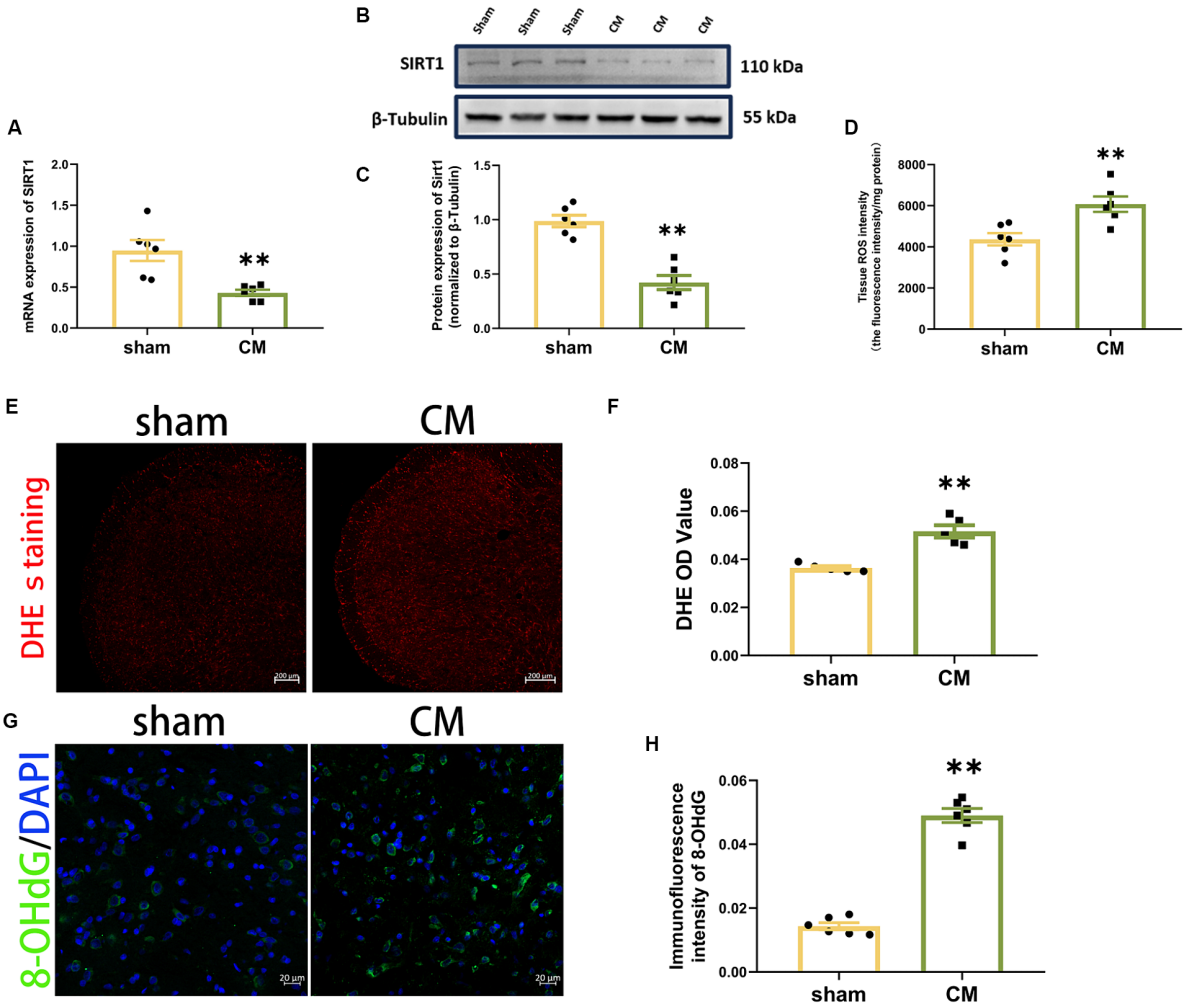
Downregulation of SIRT1 and upregulation of ROS production in CM rats. **(A)** SIRT1 expression at the mRNA level (*n* = 6/group). **(B,C)** SIRT1 expression at the protein level (*n* = 6/group). **(D)** Tissue ROS intensity in the TNC of the rats (*n* = 6/group). **(E,F)** The images of DHE staining of TNC sections and the DHE OD value of each group (Scale bars = 200 μm; *n* = 5/group). **(G,H)** The representative immunofluorescence images and quantification of 8-OHdG (Scale bars = 20 μm; *n* = 5-6/group). Two-tailed Student’s *t*-test. Data were expressed as mean ± SEM. ^*^p < 0.05, ^**^p < 0.01 vs. the Sham group.

To determine the changes in the level of SIRT1 in CM rats, the mRNA and protein expression of SIRT1 were measured separately using qRT–PCR and Western blotting analysis, respectively. As shown in Figures 2A–C, the levels of SIRT1 mRNA and protein were significantly lower in CM rats than in sham rats.

Then, we used a Tissue Reactive Oxygen Species Assay Kit and DHE staining to detect whether the production of ROS in TNC was altered in CM rats. The tissue ROS intensity and DHE OD value were significantly greater in the CM rats in comparison to sham rats (Figures 2D–F). Given that 8-OHdG can label oxidized DNA and reflect the levels of ROS (Winyard et al., 2005; Lood et al., 2016), we further examined its immunofluorescence staining. The immunosignal effect of 8-OHdG was robustly increased in the CM (Figures 2G,H). Collectively, our data show that ROS may be involved in CM.

### ROS scavenger ameliorated allodynia and reduced NR2B phosphorylation and central sensitization in CM rats

**Figure 3:**
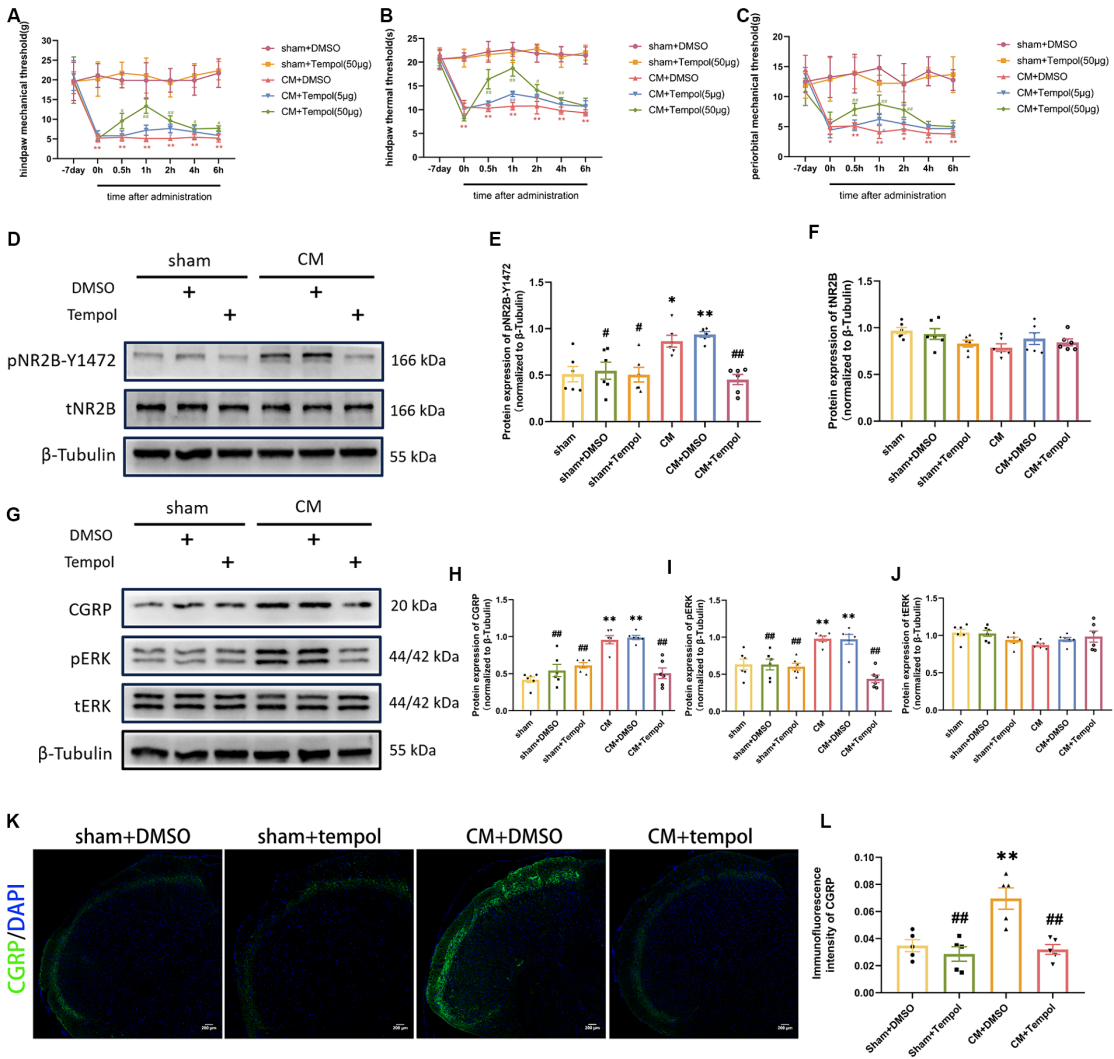
Tempol ameliorated allodynia and attenuated NR2B-Y1472 phosphorylation in CM rats. **(A–C)** The hind paw mechanical thresholds, hind paw thermal thresholds, and periorbital mechanical thresholds in each group. Two-way ANOVA with the Bonferroni *post hoc* test. Data were expressed as mean ± SEM. ^**^p < 0.01 vs. the Sham + DMSO group; *^#^p* < 0.05, *^##^p* < 0.01 vs. the CM + DMSO group; *n* = 6/group. **(D–F)** The representative western blot images and quantification of pNR2B-Y1472 and tNR2B levels (*n* = 6/group). **(G–J)** The representative western blot images and quantifications of CGRP, pERK, and tERK levels (*n* = 6/group). **(K,L)** The representative immunofluorescence images and quantification of CGRP (Scale bars = 200 μm; *n* = 5/group). One-way ANOVA with Dunnett’s *post hoc* test. Data were expressed as mean ± SEM. ^*^p < 0.05, ^**^p < 0.01 vs. the Sham group; *^#^p* < 0.05, *^##^p* < 0.01 vs. the CM group.

To explore the potential functional role of ROS in a rat model of CM, we used different doses of the ROS scavenger tempol (5 and 50 μg) to reduce ROS levels in a dose-dependent manner (Supplementary Figure 1). We found that tempol significantly attenuated allodynia in a dose-dependent manner in CM rats (Figures 3A–C). Since only the high dose of tempol significantly reduced allodynia, we proceeded with this dose. Additionally, we found that the increase in pain thresholds peaked at hour 1, so we chose to collect TNC tissue 1 h after tempol injection. However, tempol (50 μg) did not change the pain thresholds in sham rats.

As ROS are associated with allodynia in CM rats, we further explored whether the IS-induced overproduction of ROS contributed to central sensitization. We previously demonstrated that repeated IS-infusing leads to upregulation of phosphorylated NR2B-Y1472 (pNR2B-Y1472), which is an important component of synaptic plasticity and central sensitization in CM (Wang et al., 2018). We hypothesized that scavenging ROS could prevent the increase in pNR2B in CM rats. To this end, Western blot analysis was used to detect total and phosphorylated NR2B. Consistent with our previous study, the protein expression of pNR2B-Y1472, but not tNR2B, was substantially elevated in the CM (Figures 3D–F). However, the upregulation of pNR2B-Y1472 in CM rats was dramatically suppressed by the ROS scavenger tempol (Figures 3D–F). Neither pNR2B nor tNR2B in the sham group changed significantly with tempol treatment. CGRP and phosphorylated-ERK (pERK) are major markers of central sensitization in migraine (Harriott et al., 2019; Iyengar et al., 2019; Niu et al., 2020), and CGRP has been reported to be related to migraine chronicity (Cernuda-Morollon et al., 2013). Therefore, the expression levels of pERK, tERK, and CGRP in the TNC tissue of rats were also measured via western blot analysis. As shown in Figures 3G–J, the increase in CGRP and pERK protein expression in CM rats was abolished by tempol, while these differences were not observed in tERK levels. In addition, the immunofluorescence intensity of CGRP was measured using immunofluorescence analysis. We noted changes in CGRP fluorescence intensity consistent with the western blot analysis (Figures 3K,L). The above significant differences were not observed among the sham, sham + DMSO, or sham + Tempol group. Collectively, our findings suggest that ROS may be critically involved in central sensitization and allodynia in IS-induced CM rats.

### The SIRT1 agonist alleviated allodynia, reduced NR2B phosphorylation and central sensitization in CM rats

**Figure 4:**
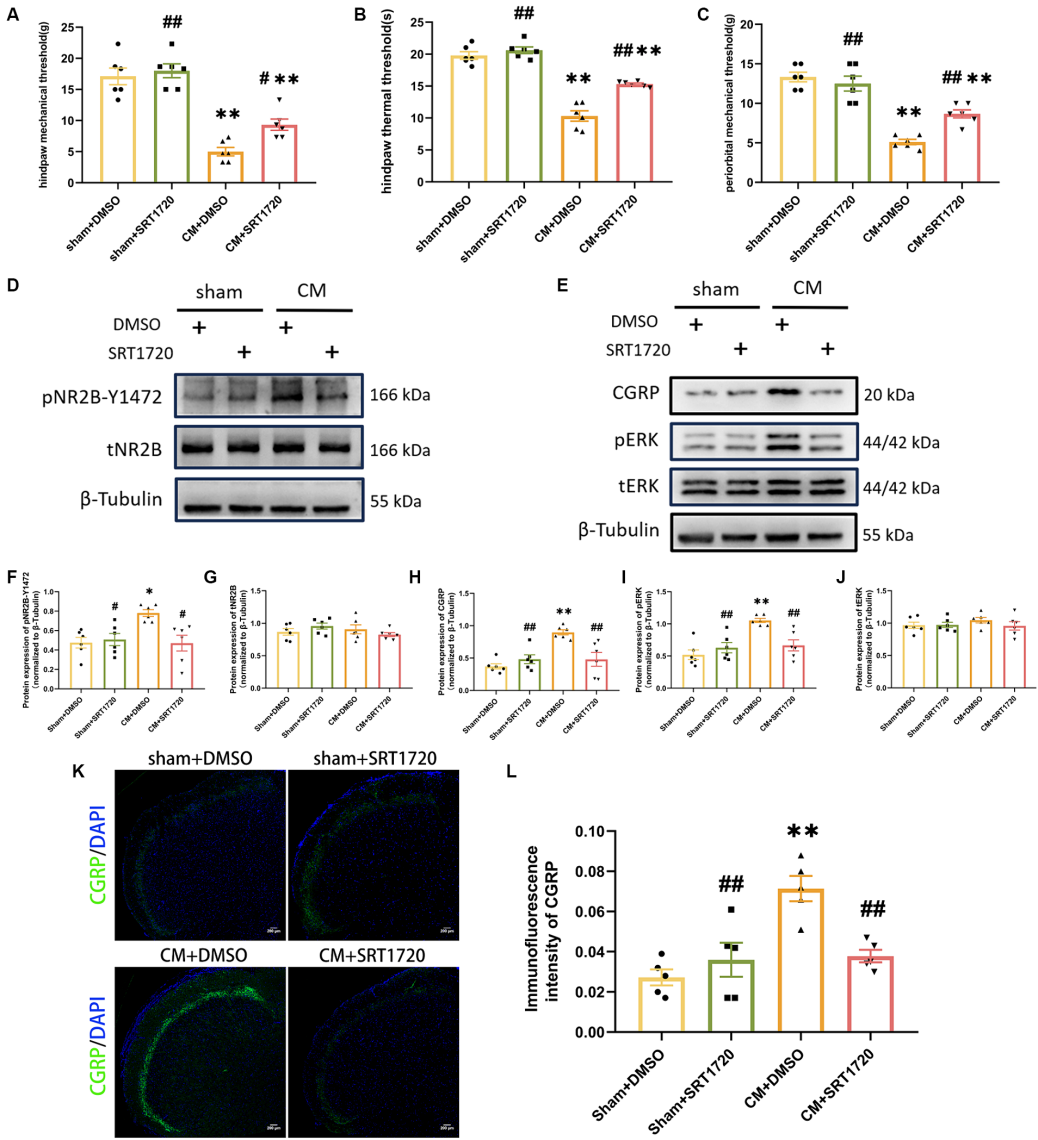
Treatment with SRT1720 ameliorated allodynia and attenuated NR2B-Y1472 phosphorylation in CM rats. **(A–C)** The hind paw mechanical thresholds, hind paw thermal thresholds, and periorbital mechanical thresholds in each group (*n* = 6/group). **(D,F,G)** The representative western blot images and quantifications of pNR2B-Y1472 and tNR2B levels (*n* = 6/group). **(E,H–J)** The representative western blot images and quantifications of CGRP, pERK, and tERK levels (*n* = 6/group). **(K,L)** The representative immunofluorescence images and quantification of CGRP (Scale bars = 200 μm; *n* = 5/group). One-way ANOVA with Dunnett’s *post hoc* test. Data were expressed as mean ± SEM. ^*^p < 0.05, ^**^p < 0.01 vs. the Sham + DMSO group; *^#^p* < 0.05, *^##^p* < 0.01 vs. the CM + DMSO group.

To investigate the function of SIRT1 in CM, we separately examined the anti-allodynic effect of SRT1720, a selective SIRT1 agonist, on sham rats and CM rats. When tested at hour 1 post-injection, SRT1720 significantly alleviated allodynia, as measured by the hind paw mechanical threshold, periorbital mechanical threshold, and hind paw thermal threshold, in CM rats (Figures 4A–C).

Having demonstrated the anti-allodynia effect of SRT1720 in CM rats, we further investigated its functional profile in pNR2B-related central sensitization in the CM model. As shown by Western blotting analysis, the relative protein level of pNR2B-Y1472 in CM + DMSO rats was increased by approximately 64% compared to that in sham + DMSO rats, but the elevated level of pNR2B-Y1472 in CM rats was markedly reduced after SRT1720 injection; however, the level of tNR2B did not significantly change (Figures 4D,F,G). Moreover, we examined the effect of the SIRT1 agonist on other indicators of central sensitization by western blotting and immunofluorescence analysis. As expected, increased levels of CGRP and pERK in CM rats were suppressed by SRT1720, while the protein expression of tERK was unaffected (Figures 4E,H–L). In addition, there were no significant differences in protein expression or pain thresholds between the sham + DMSO group and the sham + SRT1720 group.

### SIRT1 inhibitor exacerbated allodynia, increased NR2B phosphorylation, and central sensitization in CM rats

**Figure 5:**
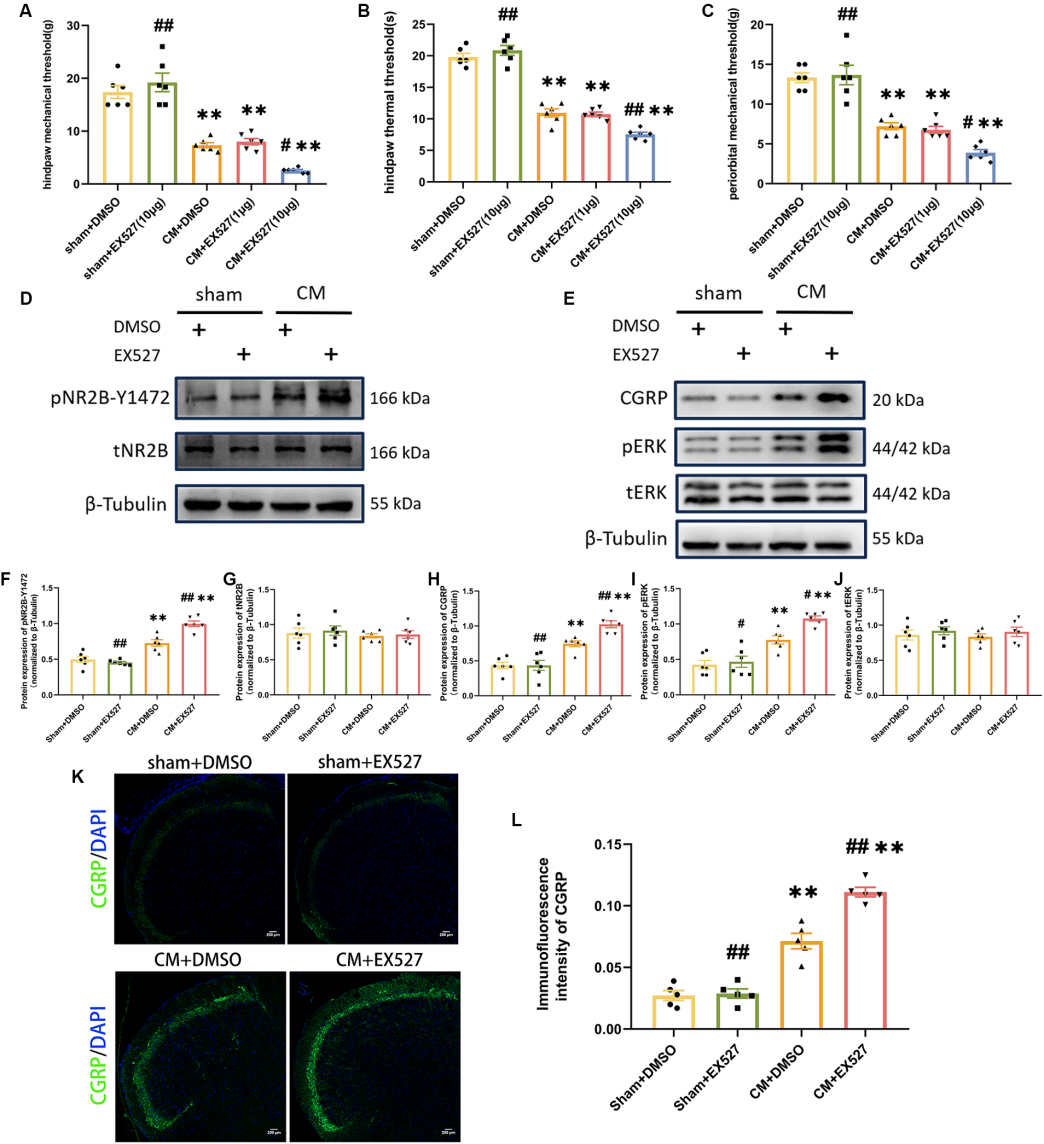
Treatment with EX527 exacerbated allodynia and aggravated NR2B-Y1472 phosphorylation in CM rats. **(A–C)** The hind paw mechanical thresholds, hind paw thermal thresholds, and periorbital mechanical thresholds in each group (*n* = 6/group). **(D,F,G)** The representative western blot images and quantifications of pNR2B-Y1472 and tNR2B levels (*n* = 6/group). **(E,H-J)** The representative western blot images and quantifications of CGRP, pERK, and tERK levels (*n* = 6/group). **(K,L)** The representative immunofluorescence images and quantification of CGRP (Scale bars = 200 μm; *n* = 5/group). One-way ANOVA with Dunnett’s *post hoc* test. Data were expressed as mean ± SEM. ^**^p < 0.01 vs. the Sham + DMSO group; *^#^p* < 0.05, *^##^p* < 0.01 vs. the CM + DMSO group.

In order to further evaluate the effect of SIRT1 on the pain threshold of CM rats, we examined whether an artificial decrease in SIRT1 expression would aggravate allodynia and increase central sensitization. For this purpose, 1 or 10 μg of EX527 (a selective SIRT1 inhibitor) or DMSO was injected into CM or sham rats following IS/PBS infusion. 1 h after EX527 administration, we observed that 10 μg EX527 showed aggravation of allodynia in CM rats but had no effect on sham rats, and the pain threshold in the CM + EX527 (1 μg) group was similar to that in the CM + DMSO group, suggesting that EX527 aggravated allodynia in a dose-dependent manner in CM rats (Figures 5A–C). We noted that EX527 further increased the expression of pNR2B-Y1472 and other central sensitization-related proteins (CGRP and pERK) in CM rats, but no significant differences were noted in the sham rats receiving EX527 or DMSO treatment (Figures 5D–L). Taken together, our results indicate the downregulation of SIRT1 may aggravate allodynia in CM rats by enhancing NR2B phosphorylation and central sensitization.

### SIRT1 agonist and inhibitor affected ROS production in CM rats

**Figure 6:**
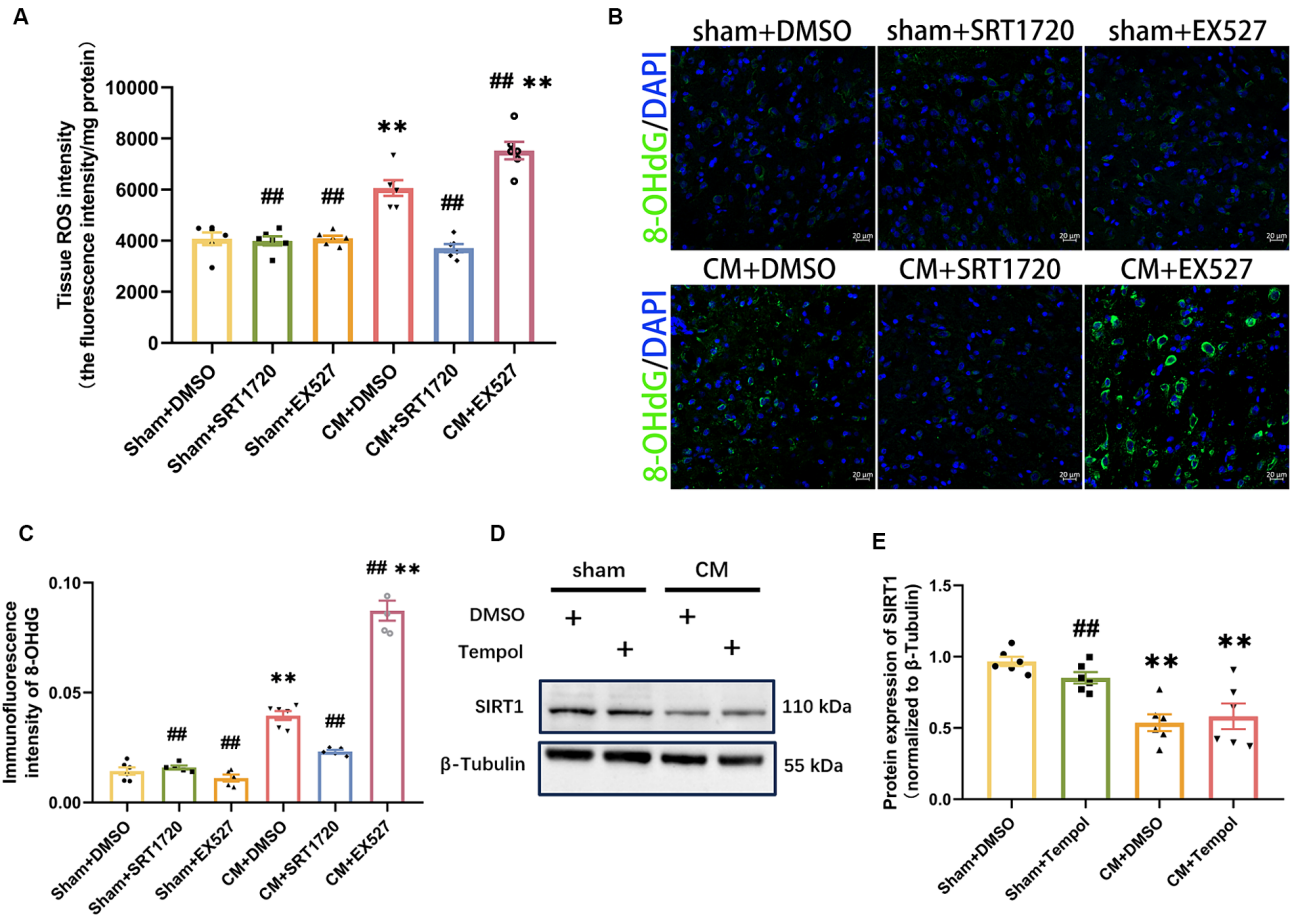
Inhibition and promotion of ROS production in CM rats treated with SRT1720 and EX527. **(A)** The tissue ROS intensity in the TNC of the rats (*n* = 6/group). **(B,C)** The representative immunofluorescence images and quantification of 8-OHdG (Scale bars = 20 μm; *n* = 5-6/group). **(D,E)** The representative western blot images and quantifications of SIRT1 levels (*n* = 6/group). One-way ANOVA with Dunnett’s *post hoc* test. Data were expressed as mean ± SEM. ^**^p < 0.01 vs. the Sham + DMSO group; *^##^p* < 0.01 vs. the CM + DMSO group.

As SIRT1 can be involved in counteracting oxidative stress, we next explored whether SIRT1 could regulate ROS levels in the CM model. ROS production was represented by the tissue ROS assay kit and 8-OHdG immunofluorescence staining analysis. The SIRT1 agonist markedly reduced the overproduction of ROS in CM rats (Figures 6A–C). Conversely, the production of ROS was greater in the CM + EX527 group than in the CM + DMSO group. However, neither SRT1720 nor EX527 had significant effects on ROS levels in the sham group. Moreover, tempol treatment did not affect the protein expression of SIRT1 in either Sham or CM rats (Figures 6D,E). Thus, these results suggest that ROS is downstream of and regulated by SIRT1 in the TNC of CM rats.

### Scavenging ROS partially reversed the negative effects of EX527 on CM rats

**Figure 7:**
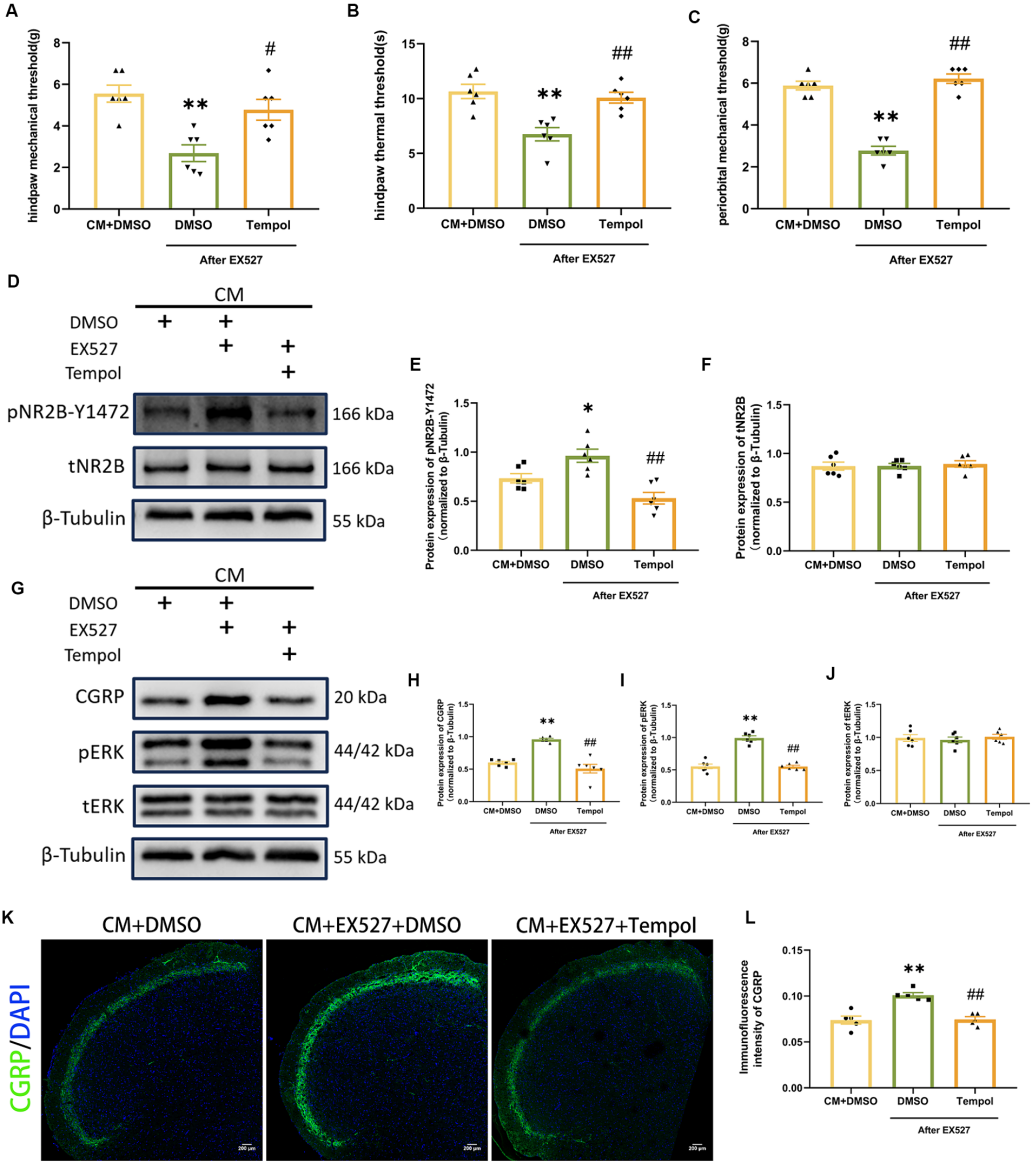
Tempol reversed the EX527-evoked exacerbation of allodynia and hyperphosphorylation of NR2B-Y1472 in CM rats. **(A–C)** The hind paw mechanical thresholds, hind paw thermal thresholds, and periorbital mechanical thresholds in each group (*n* = 6/group). **(D–F)** The representative western blot images and quantifications of pNR2B-Y1472 and tNR2B levels (*n* = 6/group). **(G–J)** The representative western blot images and quantifications of CGRP, pERK, and tERK levels (*n* = 6/group). **(K,L)** The representative immunofluorescence images and quantification of CGRP (Scale bars = 200 μm; *n* = 5/group). One-way ANOVA with Dunnett’s *post hoc* test. Data were expressed as mean ± SEM. ^*^p < 0.05, ^**^p < 0.01 vs. the CM + DMSO group; *^#^p* < 0.05, *^##^p* < 0.01 vs. the CM + EX527 + DMSO group, there was no significant difference between the CM + DMSO group and the CM + EX527 + Tempol group.

To directly investigate whether the regulatory effects of SIRT1 on pNR2B and central sensitization are mediated by ROS, we injected tempol into CM rats 1 h after EX527. Tempol reversed the significant decrease in pain threshold induced by EX527 in CM rats (Figures 7A–C). After scavenging ROS, the increase in pNR2B-Y1472 expression in the CM + EX527 group was reversed, similar to the expression of pNR2B-Y1472 expression in the CM + DMSO group (Figures 7D,E). This phenomenon was also apparent for CGRP and pERK expression (Figures 7G–I), whereas there was no significant difference in tNR2B or tERK (Figures 7F,J). Immunofluorescence staining revealed changes in CGRP levels (Figures 7K,L), which was consistent with changes in WB analysis. These findings suggest that tempol could partially inhibit the exacerbating effects of EX527 in CM rats (including allodynia and central sensitization), but not completely eliminate them.

### SIRT1 likely mediates the production of ROS via DRP1

**Figure 8:**
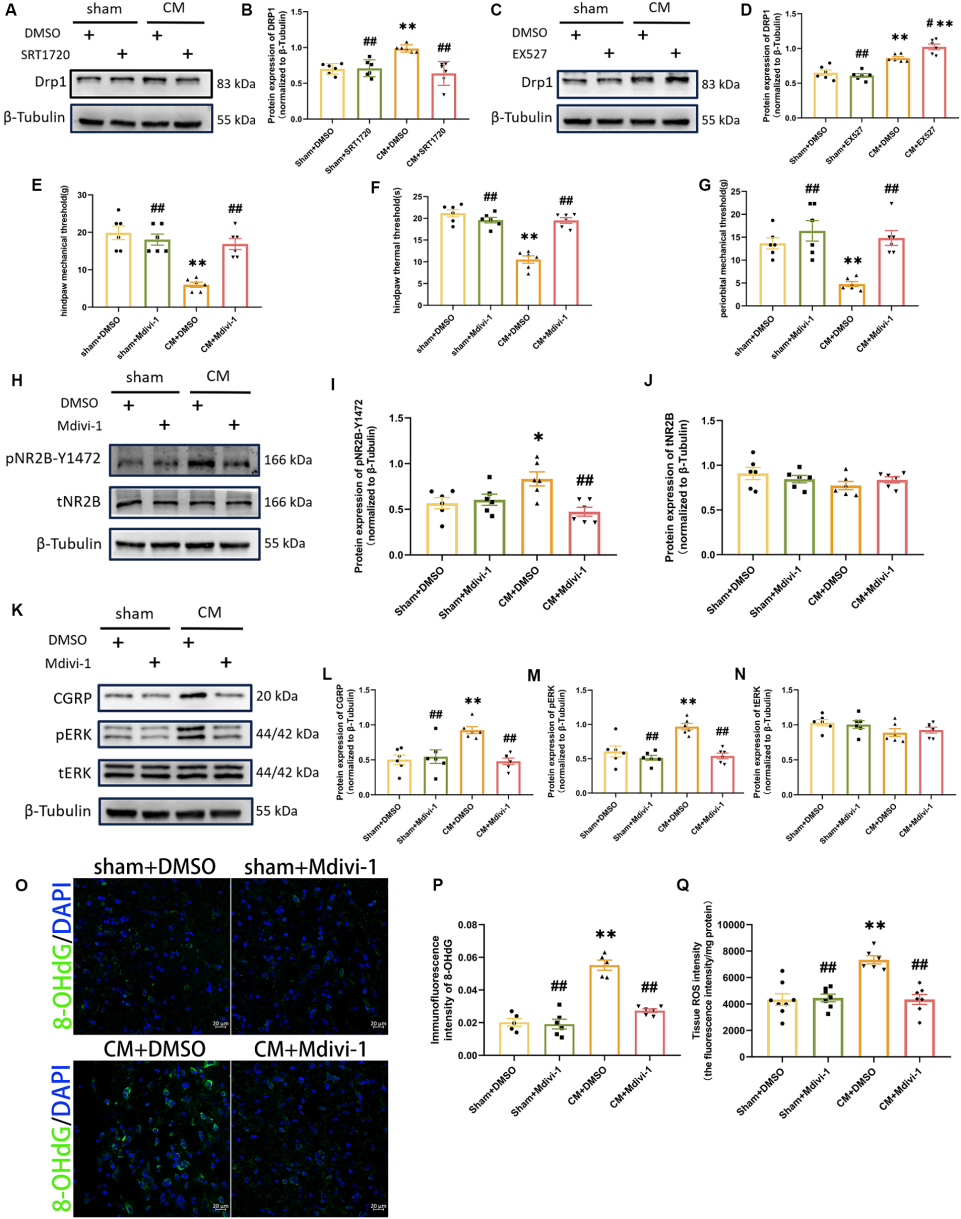
DRP1 may be partially involved in the regulation of ROS generation and NR2B-Y1472 phosphorylation by SIRT1. **(A–D)** The representative western blot images and quantification of DRP1 level (*n* = 6/group). **(E–G)** The hind paw mechanical thresholds, hind paw thermal thresholds, and periorbital mechanical thresholds in each group (*n* = 6/group). **(H–N)** The representative western blot images and quantifications of pNR2B-Y1472, tNR2B, CGRP, pERK, and tERK levels (*n* = 6/group). **(O,P)** The representative immunofluorescence images and quantification of 8-OHdG (Scale bars = 20 μm; *n* = 5–6/group). **(Q)** The tissue ROS intensity in the TNC of the rats (*n* = 6/group). One-way ANOVA with Dunnett’s *post hoc* test. Data were expressed as mean ± SEM. ^*^p < 0.05, ^**^p < 0.01 vs. the Sham + DMSO group; *^#^p* < 0.05, *^##^p* < 0.01 vs. the CM + DMSO group.

To further investigate the probable mechanism of ROS regulation by SIRT1, we focused on dynamin-related protein 1 (DRP1), a key protein involved in mitochondrial fission. By Western blotting analysis, we found that SRT1720 decreased DRP1 expression in CM rats, while EX527 had the opposite effect (Figures 8A–D). However, SRT1720 and EX527 had no significant effect on the sham group. These results suggest that SIRT1 can regulate DRP1 and may play a key role in CM. We then explored the effect of Mdivi-1, a mitochondrial fission inhibitor, on pain threshold and central sensitization. Interestingly, Mdivi-1 elevated the pain threshold in CM rats but did not have similar effects on sham rats (Figures 8E–G). Consistent with the changes in behavioral test results, the expressions of pNR2B-Y1472, pERK and CGRP, which were obviously elevated in the CM + DMSO group, were markedly reduced after Mdivi-1 treatment (Figures 8H–N). Moreover, the results of the Tissue ROS Assay Kit and the immunofluorescence results of 8-OHdG showed that Mdivi-1 could reduce ROS levels in CM rats (Figures 8O–Q). Thus, these data suggest that the regulatory role which SIRT1 has on ROS might be mediated through DRP1.

## Discussion

The remarkable contribution of our study is the proof that ROS are downregulated by the agonist SIRT1 and that the inhibition of ROS alleviates allodynia in CM rat, as well as reducing central sensitization. Here, we showed that repeated infusions of IS led to a decrease in pain threshold and allodynia in rats. We found enhanced central sensitization and increased ROS production in CM rats. We next demonstrated the contribution of ROS to central sensitization and allodynia and its regulation by SIRT1. Treatment with a SIRT1 agonist (SRT1720) reduced the levels of ROS and relieved central sensitization and allodynia in CM rats. The SIRT1 inhibitor (EX527) had the opposite effect. Interestingly, we observed that SIRT1 also regulates the expression of the mitochondrial fission protein (DRP1), and we further discovered that SIRT1 affects ROS production partially by regulating the expression of DRP1. To sum up, the data provided here highlight the important role that SIRT1 plays in the pathophysiology of CM by regulating central sensitization and allodynia through DRP1/ROS. Blocking this critical event seems to alleviate pain in CM.

There are several ways to establish an animal model of CM, including the use of nitroglycerin via intraperitoneal injection and the use of IS dural infusion (Melo-Carrillo and Lopez-Avila, 2013; Pradhan et al., 2014). The IS-induced CM model activates the trigeminal nervous system via sterile inflammation, thereby simulating the clinical symptoms of sensitization of central neurons in migraine (Strassman et al., 1996; Eikermann-Haerter and Moskowitz, 2008). Nitroglycerin provides exogenous nitric oxide (NO) to induce migraine, but NO is one of the reactive nitrogen species (RNS) that can also be involved in oxidative stress (Olesen, 2008). Therefore, to avoid the role of RNS in oxidative stress affecting the detection of ROS, a rat model of CM induced by IS was chosen for this study. Migraine is a dominant female disease, and numerous studies have confirmed sex differences in rodents with migraine (Cairns, 2007; Avona et al., 2021). The same experiment needs to be conducted on female rats with CM in the future, so as to verify the generalizability of the pathway or to explore sex differences.

Previous studies have shown that ROS, key molecules involved in oxidative stress, play pivotal roles in synaptic plasticity and neuronal excitability (Bittar et al., 2017; Esteras et al., 2022). Shan et al. (2023) reported that the levels of ROS was elevated in a mouse model of IS-induced headache. Consistent with their study, we also found increased ROS levels in a rat model of CM induced by IS. The elevated ROS levels in CM rats are in accordance with the findings of clinical studies of elevated oxidative stress indicators in the blood of migraine and CM patients (Ciancarelli et al., 2007; Lucchesi et al., 2015; Geyik et al., 2016). Of note, we only selected the TNC area for our study, and further studies on ROS levels and effects in other pain areas need to be examined in the future.

pNR2B plays an important role in synaptic plasticity and is often used in studies of cognition and pain (Yang et al., 2015; Companys-Alemany et al., 2020). A previous study reported that ROS can induce pain through the TXNIP-NLRP3-NMDAR2B pathway in type 2 diabetic rats with neuropathic pain (Wang et al., 2022). Since pNR2B-Y1472 has been previously shown to play a key role in synaptic plasticity and central sensitization in CM models (Wang et al., 2018), we further wondered whether ROS are involved in CM through pNR2B-Y1472. Tempol, a ROS scavenger, is a nontoxic molecule, and our study revealed that the use of tempol could attenuate central sensitization and allodynia by reducing the elevated pNR2B in CM rats, which is supported by the evidence that ROS modulate synaptic plasticity and central sensitization in the nervous system (Lee et al., 2007, 2012; Schwartz et al., 2008; Bittar et al., 2017; Doser et al., 2020). Furthermore, activation of pERK, a member of the MAPK family, is often used as a marker of neuronal activation and has previously been proved to play a key role in central sensitization in CM (Gao and Ji, 2009; Harriott et al., 2019; He et al., 2019). CGRP is an important neuropeptide involved in the maintenance of migraine and has been reported to be an indicator of central sensitization in CM (Goadsby et al., 2017; Niu et al., 2020). Our study also showed that the expression of central sensitization-related proteins was markedly increased in CM rats, whereas tempol treatment significantly reduced their expression. This further adds to the significant role of ROS in migraine and possibly other underlying mechanisms. However, for the ROS scavengers, we used only tempol; whether other ROS scavengers or antioxidants, such as N-tert-Butyl-α-phenylnitrone and N-acetyl-cysteine, have similar effects on the central sensitization of CM requires further exploration to determine the importance of antioxidants in the treatment of CM.

Silent information regulator 1 is a key enzyme that is considered an important cell survival protein with neuroprotective effects (Tang and Chua, 2008; Salminen et al., 2013; Manjula et al., 2020; Singh and Ubaid, 2020). We found that the use of a SIRT1 agonist (SRT1720) or a SIRT1 inhibitor (EX527) improved or exacerbated allodynia, respectively, in CM rats. These findings are consistent with previous findings showing that SIRT1 relieves a variety of chronic pain states, including neuropathic pain, bone cancer pain, and lower back pain (Zhou et al., 2017; Chen et al., 2018; Li et al., 2019; Zhang et al., 2019; Lv et al., 2021; Yang et al., 2022). In addition, we found that agonism or inhibition of SIRT1 can affect the alteration of central sensitization markers, including pNR2B-Y1472, CGRP, and pERK. SIRT1 has also been reported to be involved in oxidative stress processes *in vivo* (Singh and Ubaid, 2020; Xia et al., 2021; Zhao et al., 2021).Surprisingly, elevated or reduced SIRT1 was also shown to have a corresponding regulatory effect on ROS production in CM rats. Tempol reversed the central sensitization and allodynia exacerbated by EX527. This finding provides support that SIRT1 in the TNC could regulate allodynia by modulating ROS release. Notably, SIRT1 inhibition in sham rats did not show significant changes, and scavenging ROS only reversed the exacerbating effect of EX527 on CM rats to the level of basal CM rats, these findings indicate that SIRT1 may not play a dominant role in oxidative stress, and we cannot rule out the influence of other pathways on oxidative stress and central sensitization in CM. Although EX527 has been widely used as an inhibitor of SIRT1, its specificity *in vivo* is difficult to predict and quantify (Broussy et al., 2020), and we cannot rule out its effect on class I/II HDAC activity under different conditions. Therefore, the data obtained using EX527 solely need to be cautiously interpreted, and the role of the inactive version of EX527 or targeted knockdown of SIRT1 in the TNC in CM rats needs to be further explored in the future.

Considering the important role of SIRT1 in mitochondrial dynamics, our results suggest that SIRT1 could also modulate DRP1, a key regulatory protein of mitochondrial fission (Ding et al., 2018). Mitochondria are considered to be one of the major contributors to ROS, and a large amount of ROS can be produced by excessive mitochondrial fission (Schwartz et al., 2008; Kanda et al., 2016). We thus hypothesized that SIRT1 regulates DRP1 expression, which subsequently mediates mitochondrial fission to generate ROS. To test this hypothesis, we firstly used drugs to agonize or inhibit SIRT1, and found that the expression of DRP1 changed accordingly, indicating the regulatory effect of SIRT1 on DRP1. Treatment with the DRP1 inhibitor attenuated central sensitization and reduced ROS levels in CM rats, thereby alleviating allodynia. These findings further suggest that DRP1 plays a role in the regulation of SIRT1 and ROS in CM rats. However, the specific mechanisms of how SIRT1 modulates the expression of DRP1 in CM need to be explored further. Of note, our data contrast with those of a previous study in which upregulation of mitochondrial fission relieved neuropathic pain, possibly due to the use of different pain models (Zhang W. et al., 2022).

On the whole, our study showed that the levels of ROS were remarkably increased in the TNC in a rat model of CM and that the ROS scavenger tempol reversed the increase in the expression of pNR2B-Y1472, pERK, and CGRP, which are indicators of central sensitization, as well as allodynia, in the CM model. Furthermore, we found that (1) the SIRT1 agonist SRT1720 decreased ROS levels in the CM model, (2) the SIRT1 inhibitor EX527 exacerbated central sensitization and allodynia as well as increased ROS levels in CM rats, (3) the exacerbating effect of EX527 on central sensitization in CM rats could be reversed by tempol, (4) interfering with SIRT1 modulated the changes in Dynamin-related protein 1 (DRP1), and (5) the mitochondrial fission inhibitor Mdivi-1 suppressed the elevated ROS levels in CM rats.

## Conclusion

**Figure 9:**
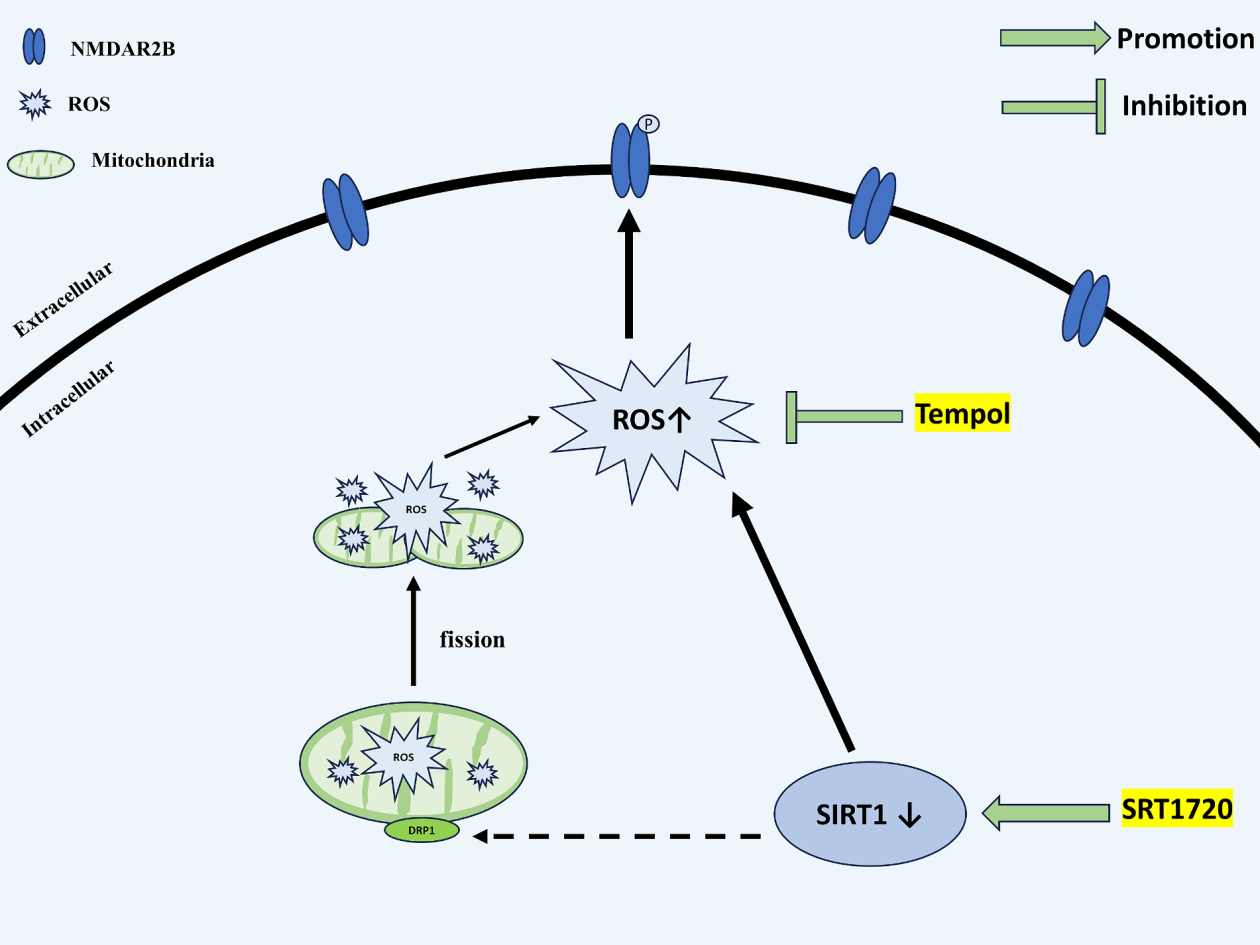
Schematic representation of the potential mechanisms. SIRT1 expression is decreased in the TNC of CM rats. The downregulation of SIRT1 increases the levels of ROS. By acting on NMDAR2B, ROS upregulate the phosphorylation of NMDAR2B-Y1472, which contributes to the central sensitization of CM. DRP1, a mitochondrial dynamics-related protein, may partially mediate the regulatory role of SIRT1 for ROS. SIRT1, Silent information regulator 1; TNC, Trigeminal nucleus caudalis; CM, Chronic migraine; ROS, Reactive oxygen species; NMDAR, N-methyl-D-aspartate receptor; and DRP1, dynamin-related protein 1.

In conclusion, as shown in Figure 9, our present study provides a possible mechanism by which agonistic SIRT1 in CM reduces central sensitization and alleviates allodynia by decreasing ROS levels, thereby decreasing the phosphorylation of NR2B-Y1472 and the expression of pERK and CGRP. In addition, SIRT1 mediates ROS production at least in part by regulating DRP1.

## Data availability statement

The original contributions presented in the study are included in the article/Supplementary material, further inquiries can be directed to the corresponding authors.

## Ethics statement

The animal study was approved by the Animal Care and Use Committee of Chongqing Medical University. The study was conducted in accordance with the local legislation and institutional requirements.

## Author contributions

XZ: Data curation, Methodology, Writing – original draft. WZ: Methodology, Writing – review & editing. YW: Methodology, Writing – review & editing. YZ: Methodology, Writing – review & editing. DZ: Methodology, Writing – review & editing. GQ: Methodology, Writing – review & editing. JZ: Writing – review & editing. LC: Supervision, Writing – review & editing.

## Supplementary material

SUPPLEMENTARY FIGURE 1 Tempol reduced ROS levels in a dose-dependent manner. One-way ANOVA with Dunnett’s *post hoc* test. Data were expressed as mean ± SEM. ^**^*p* < 0.01 vs. the Sham+DMSO group; *^##^p* < 0.01 vs. the CM + DMSO group, there was no significant difference between the CM + DMSO group and the CM + Tempol (5 μg) group, *n* = 5.
