# Supplementary figures and images for: SIRT1-regulated ROS generation activates NMDAR2B phosphorylation to promote central sensitization and allodynia in a male chronic migraine rat model
Source: Front Mol Neurosci. 2024 May 22;17:1387481. doi: 10.3389/fnmol.2024.1387481 (PMC11150646; doi:10.3389/fnmol.2024.1387481)

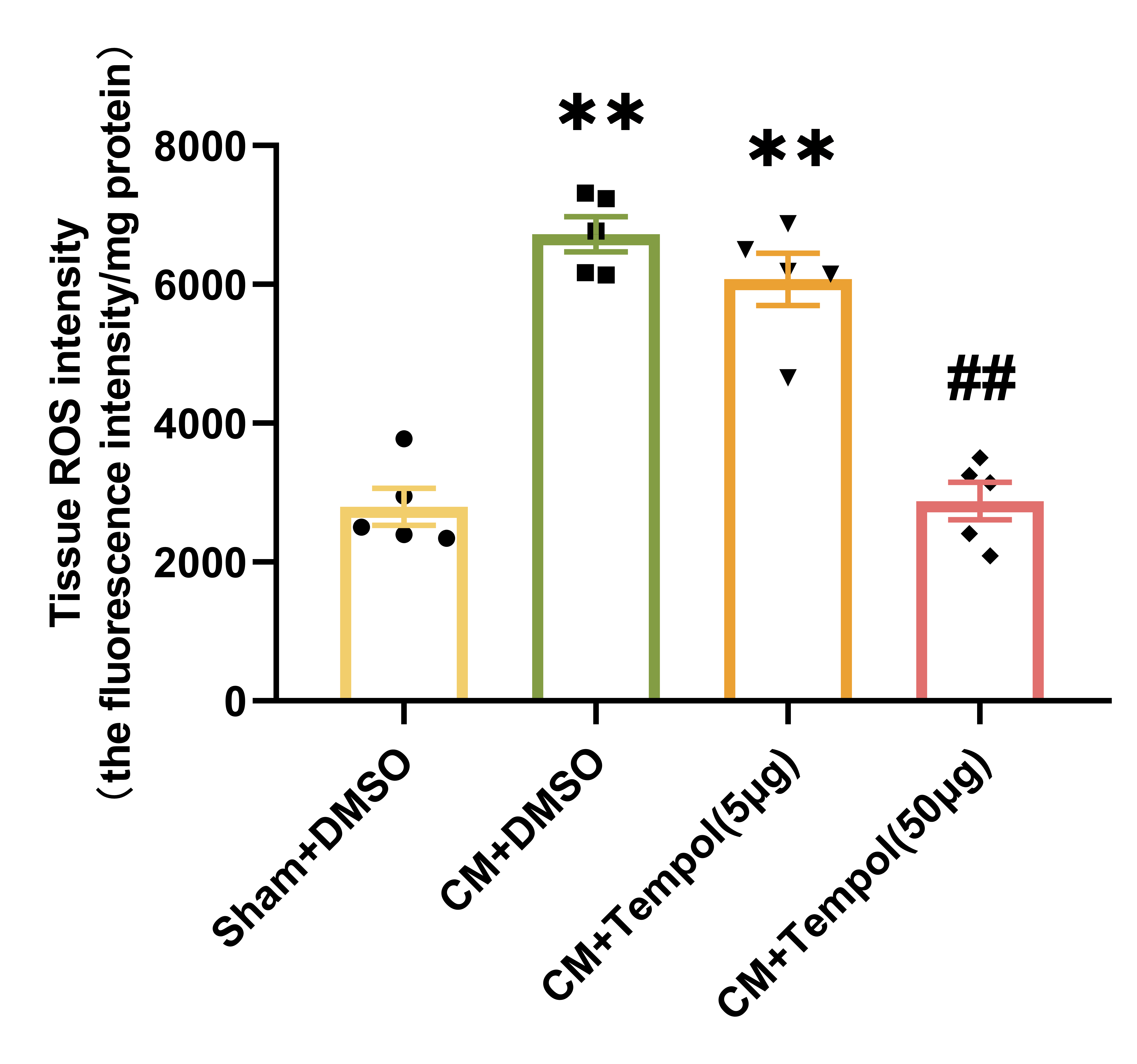

Supplement: SUPPLEMENTARY FIGURE 1 — Tempol reduced ROS levels in a dose-dependent manner. One-way ANOVA with Dunnett’s post hoc test. Data were expressed as mean ± SEM. **p < 0.01 vs. the Sham+DMSO group; ##p < 0.01 vs. the CM + DMSO group, there was no significant difference between the CM + DMSO group and the CM + Tempol (5 μg) group, n = 5. [file Image_1.TIF]
